# Validation and calibration of the patient health questionnaire (PHQ-9) in Argentina

**DOI:** 10.1186/s12888-019-2262-9

**Published:** 2019-09-18

**Authors:** María Urtasun, Federico Manuel Daray, Germán Leandro Teti, Fernando Coppolillo, Gabriela Herlax, Guillermo Saba, Adolfo Rubinstein, Ricardo Araya, Vilma Irazola

**Affiliations:** 1 South American Center of Excellence in Cardiovascular Health (CESCAS), Institute for Clinical Effectiveness and Health Policy (IECS), Buenos Aires, Argentina; 2 Social and Cardiovascular Epidemiology Research Group, School of Medicine, University of Alcala, Alcalá de Henares, Madrid, Spain; 3 Institute of Pharmacology, School of Medicine, University of Buenos Aires, Buenos Aires, Argentina; 4 National Council of Scientific and Technical Research (CONICET), Buenos Aires, Argentina; 5 Emergency Acute Inpatient Unit, “Braulio A. Moyano” Neuropsychiatric Hospital, Buenos Aires, Argentina; 6 Family Medicine Department, School of Medicine, University of Buenos Aires, Buenos Aires, Argentina; 7 Center of psychology, psychiatry and mental health Horus, Buenos Aires, Argentina; 8 Institute of criminology, National Directorate of the Federal Prison Service, Buenos Aires, Argentina; 9 Centre for Global Mental Health, London School of Hygiene and Tropical Medicine, London, UK

**Keywords:** Depression, Patient health Questionnaire-9, Mini international neuropsychiatric interview, Psychometrics, Argentina, Screening instruments

## Abstract

**Background:**

The Patient Health Questionnaire-9 (PHQ-9) is a brief tool to assess the presence and severity of depressive symptoms. This study aimed to validate and calibrate the PHQ-9 to determine appropriate cut-off points for different degrees of severity of depression in Argentina.

**Methods:**

We conducted a cross-sectional study on an intentional sample of adult ambulatory care patients with different degrees of severity of depression. All patients who completed the PHQ-9 were further interviewed by a trained clinician with the Mini International Neuropsychiatric Interview (MINI) and the Beck Depression Inventory-II (BDI-II). Reliability and validity tests, including receiver operating curve analysis, were performed.

**Results:**

One hundred sixty-nine patients were recruited with a mean age of 47.4 years (SD = 14.8), of whom 102 were females (60.4%). The local PHQ-9 had high internal consistency (Cronbach’s alpha = 0.87) and satisfactory convergent validity with the BDI-II scale [Pearson’s correlation = 0.88 (*p* < 0.01)]. For the diagnosis of Major Depressive Episode (MDE) according to the MINI, a PHQ-9 ≥ 8 was the optimal cut-off point found (sensitivity 88.2%, specificity 86.6%, PPV 90.91%). The local version of PHQ-9 showed good ability to discriminate among depression severity categories according to the BDI-II scale. The best cut off points were 6–8 for mild cases, 9–14 for moderate and 15 or more for severe depressive symptoms respectively.

**Conclusions:**

The Argentine version of the PHQ-9 questionnaire has shown acceptable validity and reliability for both screening and severity assessment of depressive symptoms.

## Background

Major Depressive Episodes (MDE) are one of the leading causes of the global disease burden [1]. In severe cases, depression can lead to suicide, which is associated with the loss of about 850,000 lives each year [2]. Mental disorders are disabling and often co-morbid with chronic physical diseases, such as cardiovascular disease [3–5].

It has been estimated that about 20% of adults in low and middle-income countries (LMIC) suffer from mental health or substance use disorder each year [6]. In Latin America, depressive disorders are the leading cause of DALYs (disability-adjusted life year) among women and the fourth cause of DALYs among men [7]. Specifically, in Argentina, the age-standardized DALY rate due to depressive disorders reached 795.7 per 100.000 in 2013 [8]. A review of epidemiological studies in general population of Argentina, Brazil, Chile, Colombia, Mexico, Peru and Puerto Rico, during the last 20 years, has shown a 12-month prevalence of major depression of 4.9% [7]. Despite its relevance to public health, depression is often unrecognized and untreated in primary care [9–11].

There is a variety of available instruments to assess depressive symptoms, but most of them have been developed in high-income countries and have not been cross-culturally adapted or validated for their use in LMIC [12]. The nine-item PHQ-9 (Patient Health Questionnaire), extensively validated in many countries, is one of the most commonly used tools for diagnosis and severity assessment of depression [13]. However, it has not been validated for its use in Argentina.

The PHQ-9 is a short, self-administered questionnaire, widely used for screening of depression in primary care settings [14], and detection of this condition in large epidemiological studies [15–18]. Because this instrument is based on DSM- IV criteria, those scoring high are often cases with Major Depressive Episode (MDE). Further, it can also be used to assess the severity of depression by identifying from mild to severe cases. However, there is growing evidence that cut-off points for determining the degree of severity may vary depending on different contexts [19–22].

Although there is a cross-culturally adapted version of the PHQ-9 in Spanish for Argentina [23], this version has not been formally validated. Additionally, the appropriate cut-off points were not ascertained to assess the severity of symptoms. Thus, the aim of this study was to validate and calibrate the PHQ-9 to determine the appropriate cut-off points to assess different degrees of severity of depression in the adult population of Argentina.

## Methods

### Participants

A cross-sectional analytical study was conducted on adults with different degrees of severity of depression as well as individuals with no depressive symptoms. The study sample was obtained between December 2013 and March 2014. Patients were recruited from two primary care clinics and two specialty mental health outpatient facilities, both from the City of Buenos Aires, Argentina. The out-patient facilities were: 1) the “Dr. Braulio A. Moyano” Hospital, which is a public neuropsychiatric hospital serving a large urban catchment area predominantly of low-income, uninsured patients; and 2) the “Foro Foundation”, a private outpatient facility treating high-income patients. The primary care clinics were: 1) The “Cooperativa de Grupo de Práctica de Medicina Familiar”, a private primary care center that treats middle-income insured patients from anywhere in Buenos Aires; and 2) the “Centro HORUS”, a private institution specialized in mental health with a multidisciplinary approach serving middle-income patients.

A purposeful quota sampling approach of persons attending these facilities was used in the study. Participants were recruited from two sources: 1) Patients referred by physicians because of the previous diagnosis of depression, and 2) patients who asked for an appointment for other health problems were approached and invited to participate. In both cases, all patients were invited to participate and asked for their signed informed consent.

Patients were recruited until the fulfillment of four quotas defined as follows: (no depression, mild, moderate, and severe symptoms of depression), according to the *Beck Depression Inventory* described below*. A* minimum of thirty patients per category was set for quota sampling.

Patients were included if they were able and willing to consent, aged 21 years or older, and were native speakers of Spanish. Exclusion criteria only applied to patients who were illiterate.

### Study instruments

#### Patient health questionnaire (PHQ-9)

We used the existing Argentinian version of the PHQ-9 instrument, which went through a full cross-cultural adaptation process [23].

This is a nine-item self-reported scale, developed to diagnose the presence and severity of depressive symptoms in primary care and the community. It is based on the DSM-IV diagnostic criteria for Major Depression Episode and it has the potential to be a dual-purpose instrument that can establish a tentative diagnosis of a depressive episode as well as depressive symptoms severity [24]. Each question in the scale has four response choices: “not at all”, “several days”, “more than half the days,” and “nearly every day.”

In the present study, we will validate and calibrate the PHQ-9 as a continuous measure.

The continuous measure is a summary score ranging from 0 to 27 and is calculated by adding up the responses to the nine questions, which allows assessing the presence and severity of a depressive episode [24]. The initial cut-off points proposed by the authors for the US population were as follows: ≥10 for diagnosis of MDE. Regarding severity, PHQ-9 comprises five categories, where a cut-off point of 0–4 indicates no depressive symptoms, 5–9 mild depressive symptoms, 10–14 moderate depressive symptoms, 15–19 moderately-severe depressive symptoms, and 20–27 severe depressive symptoms [25].

#### MINI-international neuropsychiatric interview Spanish version 5.0 (henceforth MINI): 6

The Spanish version of the Mini International Neuropsychiatric Interview (MINI) [26] was used as the gold standard for identifying the presence or absence of major depressive episode. The MINI interview is a validated tool used to diagnose minor and major depression according to the Diagnostic and Statistical Manual of Mental Disorders, Fourth Edition (DSM-IV), and is similar to the SCID (Structured Clinical Interview for DSM-IV) in operation and principle [27]. This short structured diagnostic interview explores the major Axis I psychiatric disorders in DSM-IV and ICD-10. Studies of validity and reliability have been conducted comparing the MINI to the SCID-P for DSM-III-R and CIDI (a structured interview developed by the World Health Organization for non-clinical interviewers for ICD-10). The results of these studies have shown that not only the MINI score has acceptably high validity and reliability, but also it can be administered in a much shorter period (18.7 ± 11.6 min, average 15 min) compared to the instruments mentioned above [28]. Direct clinical examination by a psychiatrist administering the Major Depressive Episode (MDE) and Dysthymia modules of the MINI was undertaken. The MDE module determined the standard diagnostic practice for the present study, while the Dysthymia module just helped us to capture the patients with lower levels of depressive symptomatology but who did not meet the MDE criteria.

#### Beck depression inventory second edition (hereafter BDI-II**)**

The locally validated version of the Beck Depression Inventory Second edition (BDI-II) was used as an instrument to ascertain symptom severity [29]. The BDI-II can be used as a self-reported questionnaire or administered by a physician. This questionnaire comprises 21 items, where each symptom is rated for the past two weeks, including the present-day on a four-point rating scale (0–3). The sum score ranges from 0 to 63. The following four severity levels are suggested: scores between 0 and 13 indicate minimal symptoms, from 14 to 19 mild, between 20 and 28 moderate, and from 29 to 63 severe symptoms of depression [29]. BDI-II has shown good psychometric properties across several settings [30, 31]. In our study, the BDI-II questionnaire was administered by trained clinicians.

We decided to address the inherent difficulty given by the fact that PHQ-9 defines five categories of depression while the BDI-II defines only four, because to our knowledge BDI-II was the unique instrument for depression screening validated in Argentina at the beginning of the study. So, in our study, the moderately severe and severe categories of the original PHQ-9 were expected to correspond to the severe category of BDI-II.

### Data collection

The PHQ-9 was self-administered, while a trained clinician conducted a structured interview (MDE, or MDE and Dysthymia modules of MINI) and applied the BDI-II questionnaire. Only those individuals who did not meet criteria for MDE received the Dysthymia module, as we wanted to ascertain how many of those classified as ‘no depressed’ could present low levels of depressive symptomatology. To minimize a possible response bias induced by the sequence of administration of the instruments, two random sequences were used as follows: a) MINI, BDI-II and PHQ-9, and b) PHQ-9, MINI, and BDI-II. All the clinicians who conducted the interviews were blinded to the results of the PHQ-9.

Additionally, we collected information on age, gender, level of education, marital status, employment, and health coverage.

### Statistical analysis

Considering expected values of sensitivity between 85 and 88%, and specificity between 92 and 95%, we calculated a minimum sample size required of 40 participants for each level of severity of depression and 30 healthy subjects with no depressive symptoms. For the sample size calculation we used the “Epidat 4.1”, free statistical software developed by Dirección Xeral de Innovación e Xestión da Saúde Pública de la Consellería de Sanidade (Xunta de Galicia) and funded by PAHO and WHO.

Criterion validity was evaluated through the comparison of the scores obtained with the PHQ-9 with the MINI interview for diagnosis, and BDI-II for the severity of depression. We calculated sensitivity, specificity and positive predictive value (PPV) and negative predictive value (NPV).

To determine the most appropriate cut-off points for PHQ-9 receiver operating characteristics (ROC) curves were generated and Youden index was calculated using the PHQ-9 summary score, where the results for depression diagnosis and severity were obtained from MINI and BDI-II respectively. All estimates were given with 95% confidence intervals.

To determine the optimal cut-off points, the area under the curve (AUC) and the PPV and NPP were evaluated and compared to the original cut-off points suggested by the authors of the original scale [25]. The AUC for different cut-off points were compared using the non-parametric statistical method described by Hanley & McNeil [32]. Youden’s index was calculated as (sensitivity + specificity – 1) [33]. The most accurate cut-off point for diagnosis and for each category of depression severity was ascertained. The Cronbach Alpha coefficient was used for measuring reliability. All data analyses were done with STATA 12.0 (StataCorp LP, College Station, TX, USA).

The data were analyzed with dysthymia cases included as “not depressed” and also excluding them to evaluate eventual changes in the results.

## Results

A total sample of 169 subjects was recruited, 102 women (60.4%) and 67 men (39.6%). The mean age was 47.4 (*SD* 14.8 years). Thirty-eight percent of them were secondary school graduates, and 14.8% were unemployed. Thirty percent of participants were married or had a partner, and 77% had social or private health insurance (Table 1). The mean BDI-II score was 21 (*SD* = 13.4) with a median score of 20 points (IQR = 19).

**Table 1:** Socio-demographic characteristics by depression severity degree of the sample of adult patients (*n* = 169)

Characteristic	Level of severity of depressive symptoms according to BDI-II				
	Overall *N* = 169	Non-depressed/Minimal symptomatology *N* = 52	Mild depressive Symptoms *N* = 32	Moderate depressive Symptoms *N* = 34	Severe depressive symptoms *N* = 51
Age, mean ± SD	47.4 ± 14.8	53.6 ± 14.8	44 ± 15.9	47 ± 14	43.7 ± 12.9
Gender, n (%)					
Male	67 (39.6%)	25 (48.1%)	13 (40.6%)	11 (32.4%)	18 (35.3%)
Female	102 (60.4%)	27 (51.9%)	19 (59.4%)	23 (67.7%)	33 (64.7%)
Level of education, n (%)					
Primary School	44 (26%)	14 (26.9%)	7 (21.9%)	8 (23.5%)	15 (29.4%)
Secondary School	65 (38.5%)	19 (36.5%)	13 (40.6%)	18 (52.9%)	15 (29.4%)
University	60 (35.5%)	19 (36.5%)	12 (37.5%)	8 (23.5%)	21 (41.2%)
Employment, n (%)					
Employed	102 (60.4%)	37 (71.2%)	17 (53.1%)	17 (50%)	31 (60.8%)
Unemployed	25 (14.8%)	0 (0%)	5 (15.6%)	9 (26.5)	11 (21.6%)
Not active	42 (24.9%)	15 (28.9%)	10 (31.3%)	8 (23.5%)	9 (17.6%)
Marital status n (%)					
Married/with partner	50 (29.6%)	22 (42.3%)	8 (25%)	8 (23.5%)	12 (23.5%)
Single/divorced/widowed	119 (70.4%)	30 (57.7%)	24 (75%)	26 (76.5)	39 (76.5%)
Health insurance n (%)	130 (76.9%)	49 (94.2%)	23 (71.9%)	27 (79.4%)	31 (60.8%)

### Criterion validity analysis for diagnosis of depression against MINI

We examined the performance of PHQ-9 against the diagnosis of MDE by MINI as the gold standard. According to MINI, 102 patients (60.36*%*) met the diagnosis of DSM-IV MDE. The mean PHQ-9 score for these patients was 14.76 (*SD* = 5.65), whereas the mean score for patients without diagnosis of MDE was 4.16 (*SD* = 4.01).

**Fig. 1:**
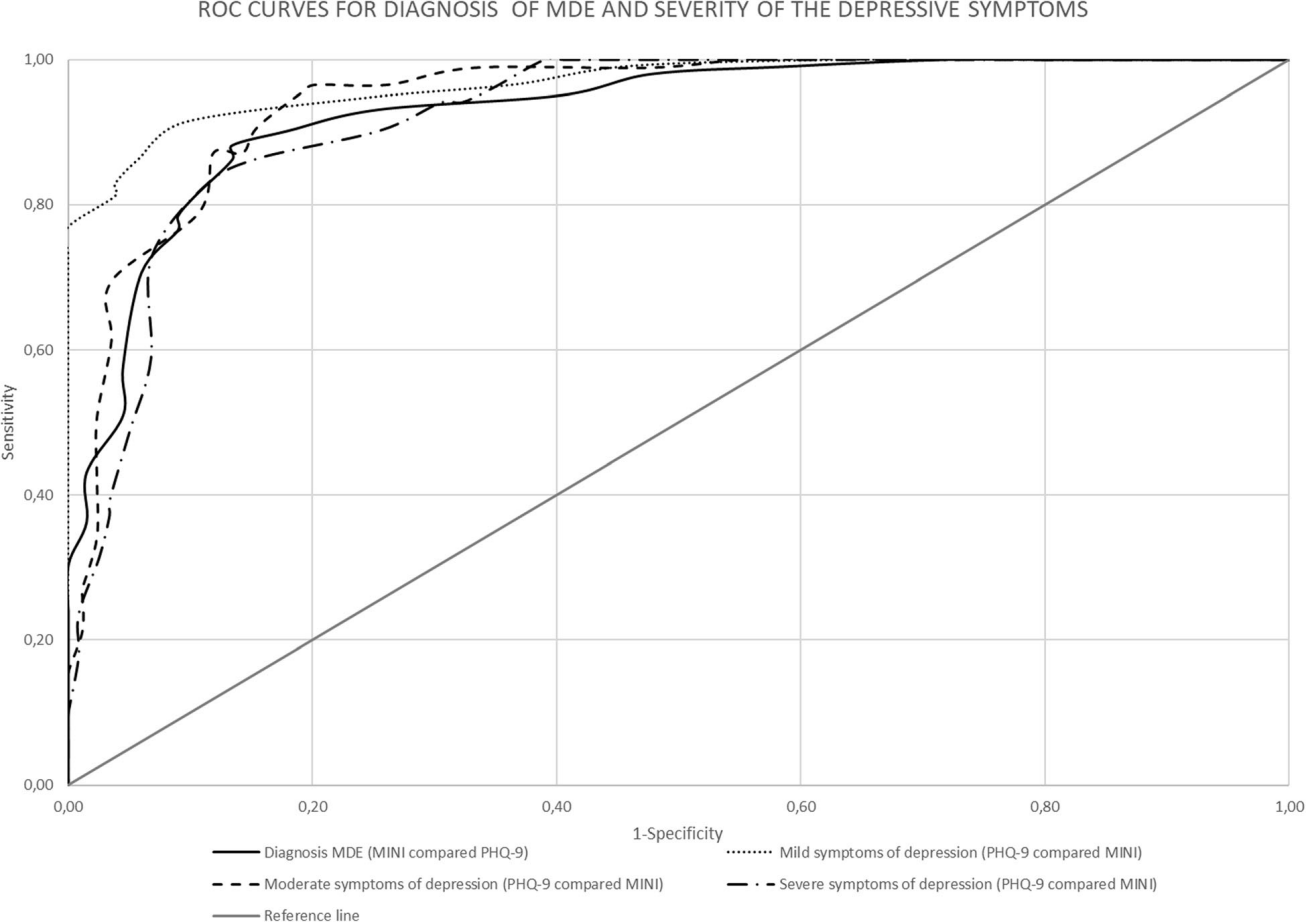
ROC Curves for the diagnosis of MDE and severity of the depressive symptoms

The validity of the PHQ-9 score as a continuous measure was also assessed. Table 2 depicts the sensitivity, specificity, PPV, NPV, and positive and negative likelihood ratio for different thresholds for diagnosing MDE against MINI. At the cut-off score of 8 or higher, the sensitivity was 88.2%, and the specificity was 86.6% (see Table 2). In addition, at this cut-off point of 8, we obtained a Youden index of J = 0.75 and 87.6*%* of subjects were correctly classified. An area under the curve (AUC) of 0.87 (95*% CI* 0.82; 0.92) also suggests good accuracy. (See Fig. 1: ROC Curve for diagnosis of MDE according to the MINI compared with the PHQ-9).

**Table 2:** Performance of PHQ-9 at different cut-off points to detect Major Depressive Episode according to MINI

PHQ-9 cutoff	Sensitivity %	Specificity %	PPV	NPV	LR+	LR-	Youden’s Index (J)
> = 6	93.14%	74.63%	84.82%	87.72%	3.67	0.09	0.68
> = 7	90.20%	82.09%	88.46%	84.62%	5.04	0.12	0.72
> = 8	88.24%	86.57%	90.91%	82.86%	6.57	0.14	0.75
> = 9	86.27%	86.57%	90.72%	80.56%	6.42	0.16	0.73
> = 10	81.37%	89.55%	92.22%	75.95%	7.79	0.21	0.71

We analyze the data with dysthymia cases included as “not depressed” first and excluding them from the analysis subsequently but the results were unaltered either way, most likely because there were only few cases (*n* = 16) of dysthymia.

**Fig. 2:**
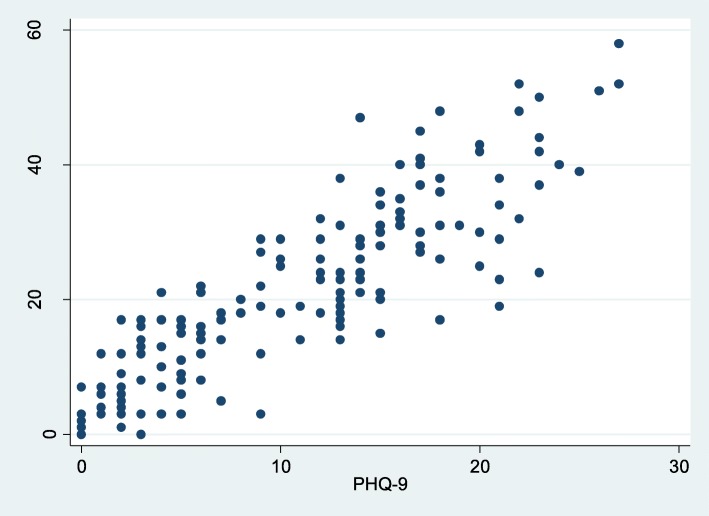
Correlation between BDI-II and PHQ-9 scores

Finally, the total score of PHQ-9 was compared with the BDI-II score. Pearson’s correlation coefficient between PHQ-9 and BDI-II was 0.88 (*p* < 0.01) indicating a positive and strong correlation between both instruments. (See Fig. 2: Correlation between BDI-II and PHQ-9 scores).

### Criterion validity analysis for depression severity assessment against BDI-II

As recommended for the Argentinean version of the BDI-II, the following categories of severity were considered: 0–13 for minimal symptoms/no depression, 14–19 for mild symptoms, 20–28 for moderate symptoms and 29–63 for severe symptoms [28].

The performance of the PHQ-9 against the different categories of severity of depressive symptoms using BDI-II as a criterion standard can be seen in Tables 3, 4, and 5. The optimal cut-off points were 6–8 for mild, 9–14 for moderate and 15 or higher for severe depressive symptoms, respectively. These thresholds showed good sensitivity, specificity, PPV, NPV, and positive and negative likelihood ratio for each category. Sensitivity ranged between 82.4% for severe symptoms to 95.3% for moderate symptoms. Specificity varied from 80.9% (moderate) to 90.4% (mild).

**Table 3:** Performance of PHQ-9 at different cut-off points in detecting *mild* symptoms of depression according to BDI-II

PHQ-9 cutoff	Sensitivity %	Specificity %	PPV	NPV	LR+	LR-	Youden’s Index (J)
> = 4	96.58%	63.46%	85.61%	89.19%	2.64	0.05	0.60
> = 5	94.87%	75.00%	89.52%	86.67%	3.79	0.07	0.70
> = 6	91.45%	90.38%	89.89%	70.09%	9.51	0.09	0.82
> = 7	86.32%	94.23%	91.80%	63.13%	14.96	0.15	0.81
> = 8	82.91%	96.15%	97.98%	71.43%	21.56	0.18	0.79

**Table 4:** Performance of PHQ-9 at different cut-off points in detecting *moderate* symptoms of depression, according to BDI-II

PHQ-9 cutoff	Sensitivity %	Specificity %	PPV	NPV	LR+	LR-	Youden’s Index (J)
> = 8	96.47%	79.76%	82.83%	95.71%	4.77	0.04	0.76
> = 9	95.29%	80.95%	83.51%	94.44%	5.00	0.06	0.76
> = 10	90.59%	84.52%	85.56%	89.87%	5.85	0.11	0.75
> = 11	87.06%	85.71%	86.05%	86.75%	6.09	0.15	0.73
> = 12	87.06%	88.10%	88.10%	87.06%	7.31	0.15	0.75

**Table 5:** Performance of PHQ-9 at different cut-off points in detecting *severe* symptoms of depression, according to BDI-II

PHQ-9 cutoff	Sensitivity %	Specificity%	PPV	NPV	LR+	LR-	Youden’s Index (J)
> = 14	86.27%	84.75%	70.97%	93.46%	5.66	0.16	0.71
> = 15	82.35%	88.98%	76.36%	92.11%	7.48	0.20	0.71
> = 16	72.55%	93.22%	82.22%	88.71%	10.70	0.30	0.66
> = 17	58.82%	93.22%	78.95%	83.97%	8.68	0.44	0.52
> = 18	49.02%	94.92%	80.65%	81.16%	9.64	0.54	0.44

High AUC estimates were also seen for all categories. AUC for mild, moderate and severe depressive symptoms was 0.91 (95% *CI* 0.86 to 0.96), 0.88 (95% *CI* 0.83 to 0.93) and 0.86 (95% *CI* 0.80 to 0.92) respectively (See Fig. 1): ROC Curve- Mild symptoms of depression with PHQ-9 compared to BDI-II; Fig. 1: ROC Curve- Moderate symptoms of depression with PHQ-9 compared to BDI-II, and Fig. 1: ROC Curve- Severe symptoms of depression with PHQ-9 compared to BDI-II).

For measuring mild symptoms of depression, a cut-off of 6 or higher showed high sensitivity (91.5%) and specificity (90.4%) and yielded a Youden index of J = 0.82 that represented 91.12% of subjects correctly classified. When comparing AUC for a cut-off point of 6 and for a cut- off point of 5 (recommended by the original authors) the difference was not statistically significant (CI overlapped). A cut-off point of 5 showed an AUC 0.85 (95% CI 0.79–0.91) whereas a cut-off point of 6 showed an AUC of 0.91 (95% CI 0.86–0.95).

Regarding moderate symptoms of depression, at the cut-off point of 9, the sensitivity was high (95.3%) but the specificity was lower but still adequate (81.0%) and the Youden index was J = 0.76. This classification yielded 88.17% of subjects correctly classified. When comparing AUC for a cut-off point of 9 and for a cut- off point of 10 (recommended by the original authors) the difference was not statistically significant (CI overlapped). A cut-off point of 9 showed an AUC of 0.88 (95% CI 0.83–0.93) whereas a cut-off point of 10 showed an AUC of 0.87 (95% CI 0.82–0.92).

Finally, the best cut-off point to measure severe depressive symptoms was 15 or higher, with a sensitivity of 82.4% and a specificity of 89.0%. Using that cut-off point, we obtained a Youden index of J = 0.71 and the PHQ-9 questionnaire correctly classified 86.98% of the subjects. In this case, as a comparison of both ROC curves for the cut-off point of 15 and the cut- off point of 20 (the recommended by original authors) a significant difference was obtained. Cut-off point of 15 showed AUC 0.86 (95% *CI* 0.80–0.92) and cut-off point of 20 showed AUC 0.67 (95% *CI* 0.60–0.74). Optimal cut-off points for the Argentinian version of PHQ-9 are shown in Table 6.

**Table 6:** PHQ-9 Scoring card for assessment of depression severity

Optimal cut-off (Original)		Optimal cut-off (For Argentina)	
Total Score	Depression Severity	Total Score	Depression Severity
0–4	None	0–5	None
5–9	Mild	6–8	Mild
10–14	Moderate	9–14	Moderate
15–19	Moderately-Severe	15–27	Severe
20–27	Severe		

Regarding internal consistency, the Cronbach’s alpha for the total PHQ-9 scale was 0.87.

## Discussion

There is a large body of evidence on PHQ-9 validation against MDE diagnosis from different countries and populations [19, 34–41]. However, there are few studies assessing calibration on severity categories [20, 21], despite a strong recommendation to explore score severity thresholds across diverse populations. [19, 22]. To our knowledge, this is the first validation and calibration study of the PHQ-9 in Argentina.

The internal consistency of PHQ-9 in this study was high and similar to the values found in other studies, which ranged from 0.67 to 0.89 [42–50]. It has been suggested that a Cronbach’s alpha of 0.70 or greater should be regarded as acceptable for a self-reported instrument [51].

When the PHQ-9 was examined for detecting MDE as a continuous measure, its validity was supported by an AUC value of 0.87, which suggests a high diagnostic accuracy. The sensitivity at the cut-off value of 8 or higher was 88.2%, and the specificity was 86.6%. These values, in particular, the specificity, are higher than those reported in two meta-analyses using PHQ-9 as a continuous measure for diagnosis of major depressive episodes [52, 53]. Furthermore, according to another recent meta-analysis, the adequate cut-off points for diagnosing MDE ranged from 8 to 11 [53]. These results, together with the cut-off point of 8 or higher suggested by another study [20] are also consistent with our results. A cut-off point of 8 showed an AUC of 0.87 (95% CI 0.82–0.93) whereas a cut-off point of 10 showed an AUC of 0.85 (95% CI 0.80–0.90). However, as expected, the sensitivity obtained with a lower threshold was higher, which becomes relevant since this instrument is intended to be used in primary care settings and population-based research.

For the present study, the MDE module of the MINI (time frame of two weeks) determined the standard diagnostic practice. While the use of Dysthymia module of the MINI (time frame of two years) just helped us to capture the patients with lower levels of depressive symptomatology but who do not reach MDE criteria. As we explained before, only those individuals who did not meet criteria for MDE received the Dysthymia module. We found that including or excluding these patients did not alter the results at all, something that was expected as there were few cases of dysthymia.

Of note, the PHQ-9 score was highly correlated with the BDI-II score. This correlation was even higher than that reported by Kneipp et al. (Pearson Correlation Coefficient = 0.80) when comparing the same instruments in low-income female populations [44]. Our results indicate a positive, strong association between both instruments, which further support the validity of the PHQ-9 measurements in this population.

Regarding the comparison of categories of severity and despite the inherent difficulty given by the fact that PHQ-9 defines five categories of depression while the BDI-II defines only four, the optimal cut-off points for the Argentine version of PHQ-9 generated the same four categories, as found in other studies (see Table 6) [11, 12]. These categories are also defined according to the DSM-IV. The thresholds for all four categories, 6–8 for mild, 9–14 for moderate and 15 or higher for severe depressive symptoms respectively showed good sensitivity, specificity, PPV, NPV, and positive and negative likelihood ratio. Of note, in the Argentinean version, the moderately severe and severe categories of PHQ-9 correspond to or could be included in the severe category of BDI-II. Since, the therapeutic approach for both, moderate-severe or severe patients, it is similar; although this misalignment of the scale categories is not ideal, it doesn’t have a relevant impact for screening or therapeutic approach purposes.

This validation study of the PHQ-9 for the Argentinean population has several strengths. First, it was rigorously designed to have an adequate representation of all the stages of severity of depression, including patients with non-depressive symptoms, which is key to ensure not only its validity for diagnosis of depression but also its calibration for different categories of severity; Secondly, we chose a criterion tool that is shorter than other diagnostic tools available in Argentina for identifying depressive cases [25], something that is important in primary care. Thirdly, the PHQ-9 is useful to assess not only the presence of clinical depression but also its degree of severity. Specifically, for severity measures, this Argentine version provides locally adjusted thresholds and follows recommendations to adapt the instrument to the context and setting where the tool is aimed to be implemented [19, 22]. Finally, its use is increasingly being valued in epidemiological research because it is brief and can be scored in a very simple way, providing a continuous measure that is easier to interpret in large epidemiological studies [15–18].

Our study presents some limitations. First, since this study has focused mainly on the city dwellers of Buenos Aires and its surroundings, its extrapolation to rural settings should be taken with caution. Yet, its usability seems to be enhanced by the fact that 90% of the Argentine population lives in urban areas. Second, the sample composition is heterogeneous with patients coming from primary and secondary care settings as well as private and state sectors. Nonetheless, this might allow us to extrapolate findings to other clinical populations. Third, we administered the instruments in a different order to avoid eventually bias, but we have not done additional analyses to ascertain if this had an impact on results. Fourth, since the PHQ-9 defines five categories of depression while the BDI-II defines only four, the optimal cut-off points for the Argentine version of PHQ-9 generated the same four categories. However, it may not have a relevant impact on screening or intervention purposes because it doesn’t condition the therapeutic approach.

## Conclusions

The Argentine version of the PHQ-9 questionnaire has shown acceptable validity and reliability for both screenings of Major Depressive Episodes and severity assessment of depressive symptoms. A definite diagnosis would ideally be attained; however, with a complementary psychiatric interview; a tool that is not always available in primary care settings. Therefore, this validated and calibrated tool could improve and facilitate the detection, classification and monitoring of depressive disorders in Argentina, particularly in the primary care setting, where depression still goes unnoticed and therefore undertreated.

## Data Availability

The datasets used and/or analyzed during the current study available from the corresponding author on reasonable request.

## Abbreviations

BDI-II: Beck Depression Inventory- II
CIDI: Composite International Diagnostic Interview
DSM-III-R: Diagnostic and Statistical Manual of Mental Disorders, Third-Research Edition
DSM-IV: Diagnostic and Statistical Manual of Mental Disorders, Fourth Edition
ICD-10: International classification of diseases. Tenth edition
MDD: Major Depressive Disorder
MDE: Major Depressive Episode
MINI: Mini International Neuropsychiatric Interview
NPV: Negative Predictive Value
PHQ-9: Patient Health Questionnaire- 9
PPV: Positive Predictive Value
ROC: Receiver Operational Curve
SCID: Structured Clinical Interview for DSM-IV
